# Dataset regarding calcium bentonite and sodium bentonite as stabilizers for roads unbound^✰^

**DOI:** 10.1016/j.dib.2022.107898

**Published:** 2022-02-02

**Authors:** Diego Maria Barbieri, Baowen Lou, Robert Jason Dyke, Hao Chen, Pengxiang Zhao, Shazim Ali Memon, Inge Hoff

**Affiliations:** a Norwegian University of Science and Technology, Department of Civil and Environmental Engineering, Høgskoleringen 7A, Trondheim, 7491, Trøndelag, Norway; b Chang'an University, School of Highway, Nan Er Huan Road (Mid-section), Xi'an, 710064, Shaanxi, China; c Oslo Metropolitan University, Department of Civil Engineering and Energy Technology, Pilestredet 35, Oslo 0166 Norway; d Xi'an University of Science and Technology, College of Safety Science and Engineering, Yanta Road 58, Xi'an, 710054, Shaanxi, China; e Nazarbayev University, School of Engineering and Digital Sciences, Civil and Environmental Engineering, Kabanbay Batyr Ave 53, Nur-Sultan, 010000, Republic of Kazakhstan

**Keywords:** Calcium bentonite, Sodium bentonite, Road stabilisation, Unbound granular materials, Repeated load triaxial test

## Abstract

The collected dataset derives from the laboratory testing of bentonite clay investigated as a stabilization technology for the unbound layers of road pavements. The effect of two kinds of bentonite (calcium based and sodium based) are assessed on two aggregate types commonly used as road construction materials. The investigation program, performed by means of repeated load triaxial tests, encompasses the different combinations of bentonite and aggregate types; two replicate specimens are tested dried for each condition. Considering the global need for ensuring well-performing road infrastructures while employing environmentally sound construction technologies, this dataset documenting the potential of bentonite clays used as road stabilizers can be of interest for several road stakeholders.

## Specification Table

**Table:** 

Subject	Civil and Structural Engineering
Specific subject area	Calcium bentonite, Sodium bentonite, Road stabilisation, Unbound granular materials, Repeated load triaxial test
Type of data	Table Image
How data were acquired	The dataset was formed by performing Repeated Load Triaxial Test (RLTT) in the laboratory.
Data format	Raw
Description of data collection	Repeated Load Triaxial Tests (RLTTs) were performed according to the standard EN 13286–7. A total of 12 samples was tested considering two types of bentonite (calcium based and sodium based) as stabilizing technologies and two types of aggregates (crushed rocks and natural gravel) as construction materials to be stabilized. 2 replicate samples were tested dried (*w* = 0%) for each combination. The deformations of the tested samples were assessed using six Linear Variable Differential Transducers (LVDTs).
Data source location	The testing campaign took place at the Department of Civil and Environmental Engineering, Norwegian University of Science and Technology (NTNU), Høgskoleringen 7A, Trondheim 7491, Norway. Aggregates came from local quarries in Trøndelag region, Norway. Bentonite clays were provided by industrial suppliers (see Acknowledgments section).
Data accessibility	Repository name: Calcium bentonite and sodium bentonite as stabilizers for roads unbound Data identification number (permanent identifier DOI number): DOI: 10.17632/9kwjrxgvmy.1 Direct link to the dataset: https://data.mendeley.com/datasets/9kwjrxgvmy/1
Related research article	D. M. Barbieri, B. Lou, R. J. Dyke, H. Chen, P. Zhao, S. A. Memon, I. Hoff. Calcium Bentonite and Sodium Bentonite as Stabilizers for Roads Unbound. Volume 6, February 2022, Cleaner Engineering and Technology. https://doi.org/10.1016/j.clet.2021.100372

## Value of the Data

• In light of the significant global need for efficient and sustainable construction and maintenance of road pavement infrastructures, the dataset is useful to appraise the stabilization potential of two types of bentonite clay (calcium based and sodium based).
• As road pavements are an important infrastructural asset for each nation's economy, the employment of green efficient technologies for road stabilization is relevant for several stakeholders such as researchers, engineers, professionals, entrepreneurs and agencies.
• The dataset can be used to quantify the stabilization potential attained by bentonite clay in roads unbound. The experimental data can be analysed according to several models to appraise resilient modulus and deformation properties.
• The rock aggregates tested in the investigation campaign have been selected as they are largely used in the central part of Norway as road construction materials.

## 1 Data Description

The dataset is collected during an experimental testing campaign assessing the stabilization potential of bentonite clay for roads unbound [1]. Considering the huge extent of the global road network and the associated need for efficient and sustainable construction and maintenance operations [2,3], several technologies can be used to improve the mechanical properties of road unbound layers [4], [5], [6]. Being the application of bentonite an environmentally sound solution for civil construction purposes [7,8], two types of bentonite clays are considered, namely Calcium based Bentonite (CaB) and Sodium based Bentonite (NaB). They are applied on two different kinds of aggregates commonly used for road construction, namely Crushed Rock Aggregates (RCA) and Natural Gravel Aggregates (NGA). The aggregates are tested both treated with bentonite clays and untreated (Unbound Granular Material, UGM). The investigation programme is undertaken by means of Repeated Load Triaxial Test (RLTT). The formed dataset is composed by raw data and pictures of the specimens (https://data.mendeley.com/datasets/9kwjrxgvmy/1).

The experimental RLTT data contained in the folder “Data of Repeated Load Triaxial Test” are arranged in 6 subfolders as reported in Table 1. Two replicate samples (denominated “01” and “02”) are tested dried for each combination and the information available for each specimen are one spreadsheet with raw data (.xlsx) and two pictures (.jpg). The amount of dry bentonite present in each sample is 0.4% in mass and it is applied at the Optimum Moisture Content (OMC) of the chosen particle size distribution *w* = 5% [9].

**Table 1:** Subfolders names and corresponding RLTT investigated combinations.

Numbering	Subfolder name	Aggregate type	Bentonite type
01	CRA-UGM	crushed rock	–
02	CRA-CaB	crushed rock	calcium based
03	CRA-NaB	crushed rock	sodium based
04	NGA-UGM	natural gravel	–
05	NGA -CaB	natural gravel	calcium based
06	NGA -NaB	natural gravel	sodium based

The content of all the spreadsheets is arranged according to a consistent logic [10]. The five worksheets contained in each spreadsheet are denominated “Sequence 1”, “Sequence 2”, “Sequence 3”, “Sequence 4”, “Sequence 5”, which correspond to as many loading sequences forming one RLTT. Column A reports the number of the loading steps (each sequence comprises up to six steps), while column B, C, D and E display information about time *t*, temperature *T*, deviatoric pulse number and frequency *f*, respectively. The dynamic part (*σ_d,dyn_*) and the static part (*σ_d,st_*) of the deviatoric stress *σ_d_* are reported in columns F and G, while, similarly, the dynamic part (*σ_t,dyn_*) and the static part (*σ_t,st_*) of the triaxial stress *σ_t_* are specified in columns H and I. Six Linear Variable Displacement Transformers (LVDTs) measure the specimen deformation considering the axial elastic components (*ε_a,el,01_, ε_a,el,02_, ε_a,el,03_* listed in columns J, L, N), axial plastic components (*ε_a,pl,01_, ε_a,pl,02_, ε_a,pl,03_* listed in columns K, M, O), radial elastic components (*ε_r,el,01_, ε_r,el,02_, ε_r,el,03_* listed in columns P, R, T) and radial plastic components (*ε_r,pl,01_, ε_r,pl,02_, ε_r,pl,03_* listed in columns Q, S, U).

**Fig. 1:**
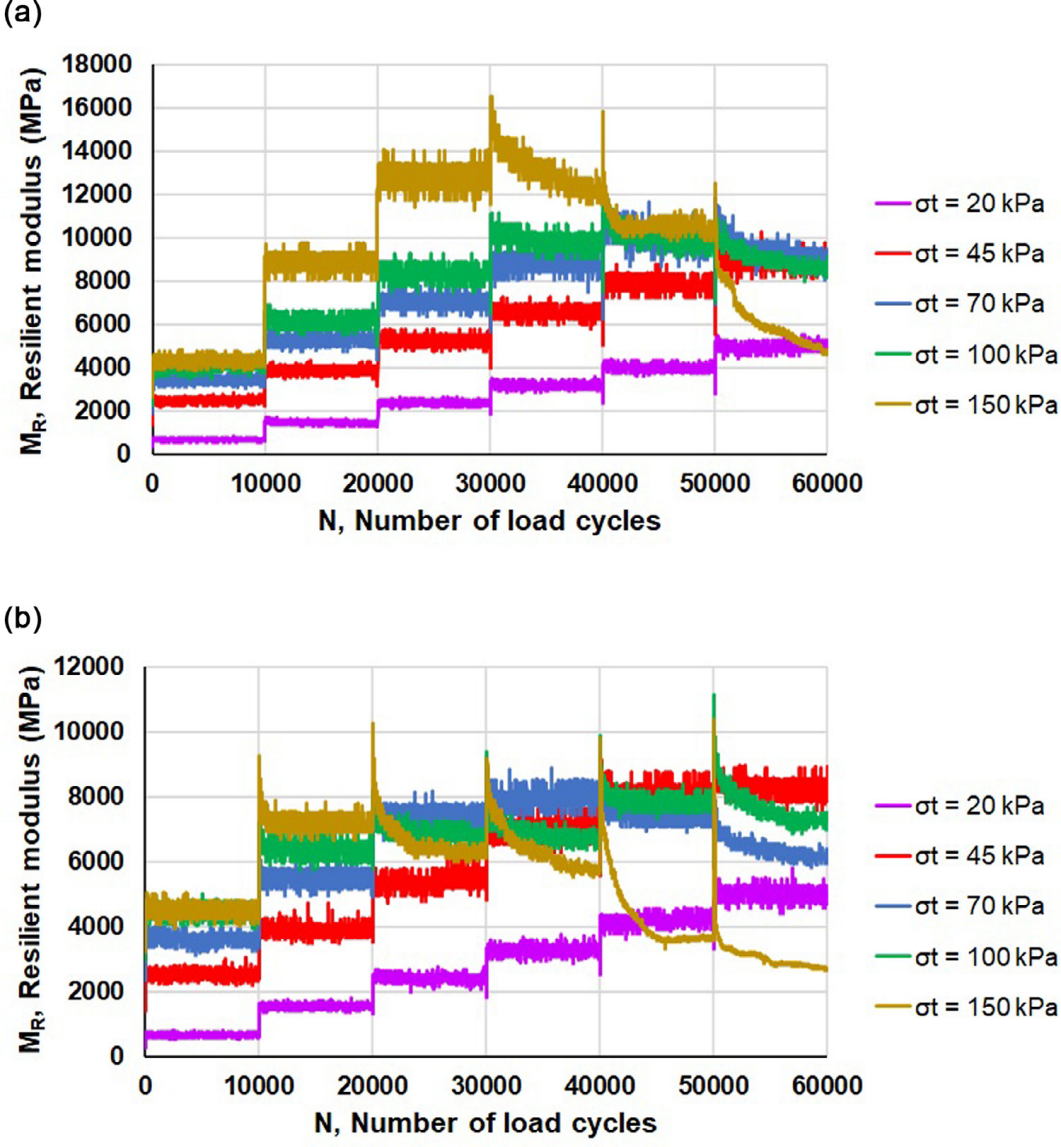
Resilient modulus *M_R_* of “CRA-CaB” replicate specimens: sample 01 (a) and sample 02 (b).

**Fig. 2:**
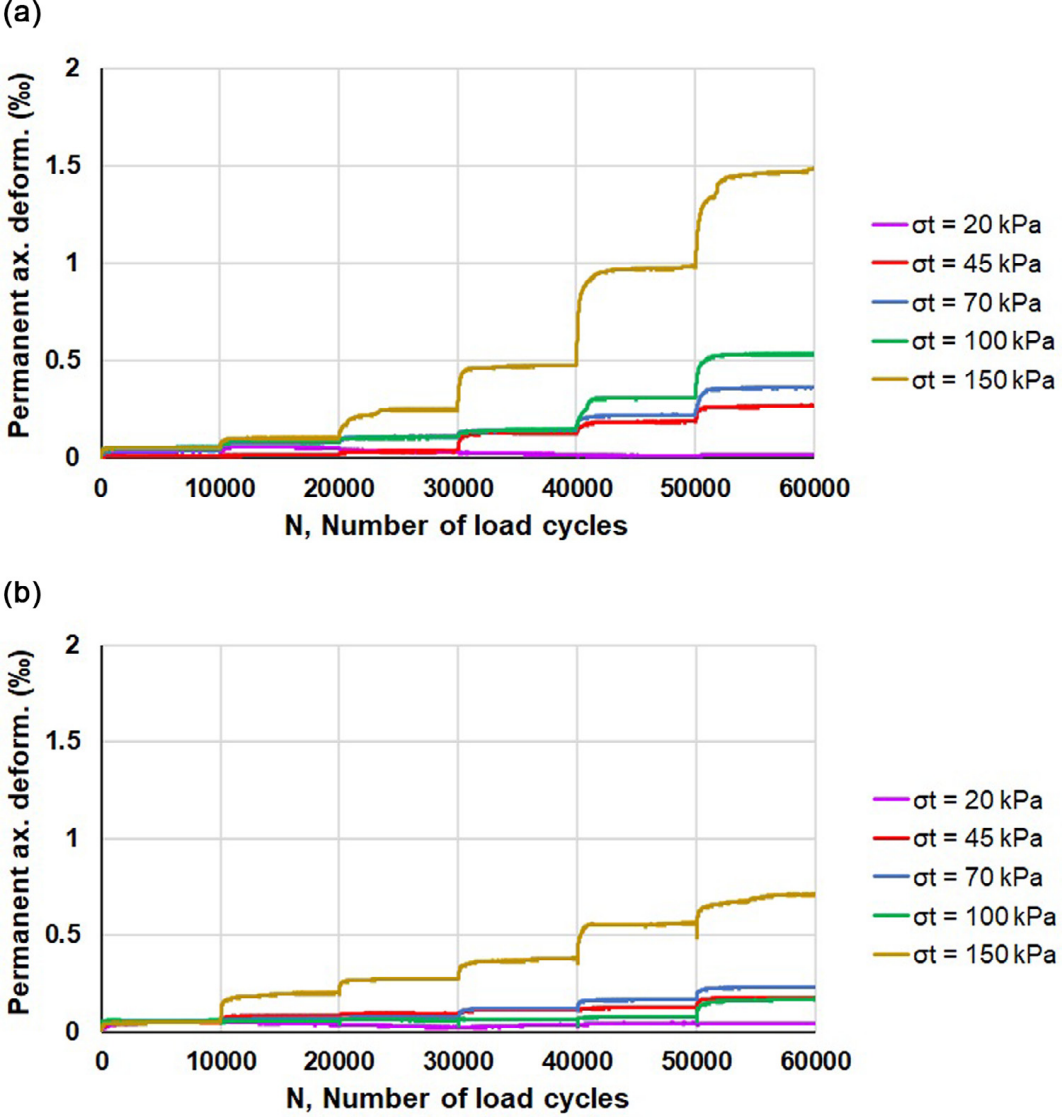
Axial plastic deformation of “CRA-CaB” replicate specimens: sample 01 (a) and sample 02 (b).

The main mechanical properties that are directly relevant to road pavement engineering and can be assessed by means of RLTTs are elastic stiffness (resilient modulus, *M_R_*) and the resistance against permanent. As an example, considering crushed rock aggregates stabilized with calcium based bentonite, Figs. 1 and 2 depict the experimental values of *M_R_* and axial plastic deformation, respectively, as a function of the number of load cycles *N*. Furthermore the trend of the experimental data can be determined considering the several regression models available in literature [11], [12], [13], [14], [15], [16].

## 2 Experimental Design, Materials and Methods

Both the crushed rock and natural gravel aggregates were derived from local quarries in Trøndelag region, Norway. They have been selected as they are largely used as construction materials in both unbound and bound layers of road pavements realized in the central part of the country [17]. The two types of calcium based and sodium based bentonite clays were supplied by industrial producers. The overarching goal of the research was to investigate environmentally friendly technologies that can be used for the construction or stabilization of road unbound layers [18,19]; in this regard, the application of bentonite is still relatively unexplored [20], [21], [22].

**Fig. 3:**
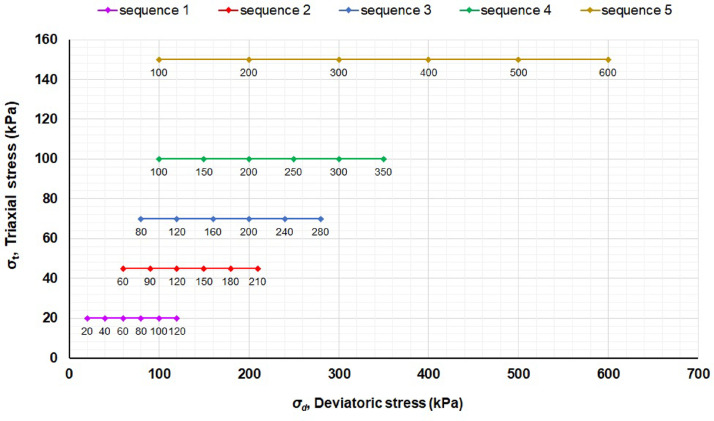
Values of triaxial stress *σ_t_* and deviatoric stress *σ_d_* defining the MS LSL RLTT.

The research activities were accomplished performing RLTTs according to Multi-Stage Low Stress Level (MS LSL) indicated in the CEN standard “13286–7 Cyclic load triaxial test for unbound mixtures” [23]. A RLTT comprised thirty loading steps, where each of them referred to a precise combination of deviatoric stress *σ_d_* and triaxial stress *σ_t_* as illustrated in Fig. 3: the former one was applied according to a sinusoidal pattern using a hydraulic jack, while the latter one was applied by pressurized water. Given a constant value of *σ_t_* and a dynamic deviatoric stress *Δσ_d,dyn_*, the resilient modulus *M_R_* is defined as

(1) $$M_{R}=\frac{\Delta \sigma_{d,dyn}}{\varepsilon_{a,el}},$$

with *ε_a,el_* the average axial resilient strain evaluated by the three axial LVDTs.

## CRediT authorship contribution statement

**Diego Maria Barbieri:** Conceptualization, Methodology, Software, Validation, Formal analysis, Investigation, Resources, Data curation, Writing – original draft, Visualization, Project administration. **Baowen Lou:** Conceptualization, Methodology, Software, Validation, Formal analysis, Investigation, Resources, Data curation, Writing – original draft. **Robert Jason Dyke:** Conceptualization, Methodology, Formal analysis, Investigation, Resources, Data curation, Writing – review & editing. **Hao Chen:** Investigation, Resources, Writing – review & editing, Visualization. **Pengxiang Zhao:** Writing – review & editing, Visualization. **Shazim Ali Memon:** Writing – review & editing, Visualization, Supervision. **Inge Hoff:** Conceptualization, Methodology, Writing – review & editing, Visualization, Supervision, Project administration, Funding acquisition.

## Declaration of Competing Interest

This work was supported by Norwegian Public Roads Administration (VegDim project, grant number 605377) and by Research Council of Norway (HERMES project, grant number 299538).

## Appendix Supplementary materials

Image, application 1

Image, application 2
