# Supplementary material for: Dataset regarding calcium bentonite and sodium bentonite as stabilizers for roads unbound
Source: Data Brief. 2022 Feb 2;41:107898. doi: 10.1016/j.dib.2022.107898 (PMC8844401; doi:10.1016/j.dib.2022.107898)

To:

Yolanda Picó, *Data in Brief, Editor-in-Chief*

Nicholas A. Pullen, *Data in Brief, Editor-in-Chief*

Trondheim, Norway, November 22^th^, 2021

Dear Drs. Picó and Pullen

We acknowledge that the submission declaration of Data in Brief has been complied with. We also confirm that all necessary permissions have been obtained. The authors declare that there is no conflict of interest regarding the publication of this article.

Sincerely yours,

*Diego Maria Barbieri,*

*also on behalf of all co-authors*


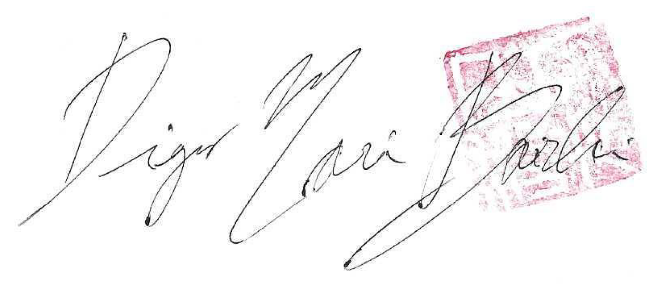

Supplement: Supplementary file 1 [file mmc1.docx]
