# Supplementary material for: Dataset regarding calcium bentonite and sodium bentonite as stabilizers for roads unbound
Source: Data Brief. 2022 Feb 2;41:107898. doi: 10.1016/j.dib.2022.107898 (PMC8844401; doi:10.1016/j.dib.2022.107898)
Supplement: Supplementary file 2 [file mmc2.docx]

**Diego Maria Barbieri**

Conceptualization, Methodology, Software, Validation, Formal analysis, Investigation, Resources, Data curation, Writing - Original Draft, Visualization, Project administration

**Baowen Lou**

Conceptualization, Methodology, Software, Validation, Formal analysis, Investigation, Resources, Data curation, Writing - Original Draft

**Robert Jason Dyke**

Conceptualization, Methodology, Formal analysis, Investigation, Resources, Data curation, Writing - Review & Editing

**Hao Chen**

Investigation, Resources, Writing - Review & Editing, Visualization

**Pengxiang Zhao**

Writing - Review & Editing, Visualization

**Shazim Ali Memon**

Writing - Review & Editing, Visualization, Supervision

**Inge Hoff**

Conceptualization, Methodology, Writing - Review & Editing, Visualization, Supervision, Project administration, Funding acquisition
